# Regional Cerebellar Volume Loss Predicts Future Disability in Multiple Sclerosis Patients

**DOI:** 10.1007/s12311-021-01312-0

**Published:** 2021-08-21

**Authors:** Katrin Parmar, Vladimir S. Fonov, Yvonne Naegelin, Michael Amann, Jens Wuerfel, D. Louis Collins, Laura Gaetano, Stefano Magon, Till Sprenger, Ludwig Kappos, Cristina Granziera, Charidimos Tsagkas

**Affiliations:** 1 Neurologic Clinic and Policlinic, Departments of Medicine, Clinical Research and Biomedical Engineering, University Hospital Basel and University of Basel, Basel, Switzerland; 2 Translational Imaging in Neurology (ThINk) Basel, Department of Medicine and Biomedical Engineering, University Hospital Basel and University of Basel, Basel, Switzerland; 3 Reha Rheinfelden, Rheinfelden, Switzerland; 4 McConnell Brain Imaging Center, Montreal Neurological Institute, McGill University, Montreal, QC, CA USA; 5 Medical Image Analysis Center (MIAC AG), Basel, Switzerland; 6 Quantitative Biomedical Imaging Group (Qbig), Department of Biomedical Engineering, University of Basel, Basel, Switzerland; 7 Neuroscience/Digital Medicine, F. Hoffmann-La Roche Ltd, Basel, Switzerland; 8 Roche Pharma Research and Early Development, Roche Innovation Center Basel, Basel, Switzerland; 9 Department of Neurology, DKD HELIOS Klinik Wiesbaden, Wiesbaden, Germany

**Keywords:** Multiple sclerosis, Magnetic resonance imaging, Cerebellum and atrophy

## Abstract

Cerebellar symptoms in multiple sclerosis (MS) are well described; however, the exact contribution of cerebellar damage to MS disability has not been fully explored. Longer-term observational periods are necessary to better understand the dynamics of pathological changes within the cerebellum and their clinical consequences. Cerebellar lobe and single lobule volumes were automatically segmented on 664 3D-T1-weighted MPRAGE scans (acquired at a single 1.5 T scanner) of 163 MS patients (111 women; mean age: 47.1 years; 125 relapsing–remitting (RR) and 38 secondary progressive (SP) MS, median EDSS: 3.0) imaged annually over 4 years. Clinical scores (EDSS, 9HPT, 25FWT, PASAT, SDMT) were determined per patient per year with a maximum clinical follow-up of 11 years. Linear mixed-effect models were applied to assess the association between cerebellar volumes and clinical scores and whether cerebellar atrophy measures may predict future disability progression. SPMS patients exhibited faster posterior superior lobe volume loss over time compared to RRMS, which was related to increase of EDSS over time. In RRMS, cerebellar volumes were significant predictors of motor scores (e.g. average EDSS, T25FWT and 9HPT) and SDMT. Atrophy of motor-associated lobules (IV-VI + VIII) was a significant predictor of future deterioration of the 9HPT of the non-dominant hand. In SPMS, the atrophy rate of the posterior superior lobe (VI + Crus I) was a significant predictor of future PASAT performance deterioration. Regional cerebellar volume reduction is associated with motor and cognitive disability in MS and may serve as a predictor for future disease progression, especially of dexterity and impaired processing speed.

**Supplementary Information:**

The online version contains supplementary material available at 10.1007/s12311-021-01312-0.

## Introduction

Measures of central nervous system (CNS) atrophy are increasingly recognized as viable biomarkers of disease burden in multiple sclerosis (MS) [1, 2]. While the occurrence of cerebellar signs and symptoms in MS has been well-known [3], the exact contribution of cerebellar damage to disability in MS has still not been fully explored.

The cerebellum is known to play an important role in motor function, coordination and cognitive-behavioural processing [4, 5]. In MS patients, both cerebellar signs and symptoms are significant contributors to the development of disability and often progress despite disease-modifying treatment [6, 7]. Neuropathological studies have shown extensive demyelination in the cerebellar cortex mainly of progressive MS patients [8]. Further cross-sectional neuroimaging studies have confirmed cerebellar volume reductions in patients with MS when compared to healthy controls [9–13]. However, it is currently not clear to what extent atrophy in the cerebellum matters clinically. Also, data from previous MRI studies on cerebellar volume reductions, especially early in the disease course, provided inconsistent findings [10, 14], and the clinical correlations of volume abnormalities were limited. While correlation with the expanded disability status scale (EDSS) was at best modest [10] or non-existing [11, 15, 16], more convincing correlations were observed between cerebellar volumes and clinical measures, which directly reflect fine-motor skills, locomotion or cognition [9, 12–14, 17]. The high variability between studies may be explained by the application of different techniques (semi-automated versus automated segmentation, voxel based morphometry, whole versus lobule-wise analyses etc.), heterogeneity in patient cohorts (disease duration, disease types, number of patients) and the fact that mainly cross-sectional study designs were used.

Longer-term observational periods are necessary to better understand the dynamics of pathological changes within the cerebellum and their clinical consequences in MS patients. We therefore analysed clinical and MRI data of a large cohort of patients with relapse-onset MS with up to 11 years of follow-up in order to examine the relationship of cerebellar volume loss and disease progression, and determine whether cerebellar atrophy may serve as a potential biomarker for future disease progression. We applied our in-house developed *‘Rapid Automatic Segmentation of the human Cerebellum And its Lobules’* (*RASCAL)* [18], showing high accuracy in a previous segmentation challenge (Carass et al. 2018).

## Methods

### Participants and Study Design

Clinical and MRI data of 163 MS patients, 125 with relapsing–remitting (pwRRMS) and 38 with secondary progressive disease courses (pwSPMS), of an ongoing large-scale cohort study from a single centre (tertiary MS Centre, University Hospital, Basel) were analysed retrospectively (for details see Tables 1 and 2). Patients were followed annually including a clinical visit and MRI over a median of 4 years (here referred to as: period I) plus a clinical long-term follow-up of a median of 6 years (here referred to as: period II). The diagnosis of MS was made in accordance with international panel established criteria [19].

**Table 1:** Baseline demographics

	Total	RRMS	SPMS	*p*-level
	*n* = 163	*n* = 125	*n* = 38	
Age years (mean ± SD)	47.1 ± 11.3	44.7 ± 10.9	55.1 ± 8.8	< 0.001
sex (f:m)	111: 52	90:35	21:17	0.08
Disease duration years (mean ± SD)	16.1 ± 9.4	14.5 ± 10.9	21.5 ± 9.7	< 0.001
Phase I follow-ups (median (range))	4 (1–6)	5 (1–6)	4 (1–6)	0.76
Phase II follow-ups (median (range))	6 (2–8)	6 (2–8)	5 (2–7)	0.05
EDSS (median (range))	3.0 (0–7.5)	2.5 (0–6.5)	5.25 (3–7.5)	< 0.001
Pyramidal FSS (median (range))	2.0 (0–4)	1.0 (0–4)	3.0 (1–4)	< 0.001
Sensory FSS (median (range)	2.0 (0–4)	1.0 (0–4)	2.0 (0–4)	< 0.001
Cerebellar FSS (median (range))	1.0 (0–4)	1.0 (0–4)	3.0 (1–4)	< 0.001
T25FWT sec (median (range))	4.95 (2.25–82.2)	4.6 (2.25–30.2)	8.95 (2.25–82.2)	< 0.001
9HPT DH sec (median (range))	19.7 (13.8–94)	18.9 (13.8–74.7)	24.5 (16.5–93.9)	< 0.001
9PHT NDH sec (median (range))	20.8 (13.6–167.5)	20 (13.6–114)	26.8 (19.9–167.5)	< 0.001
SDMT median score (range)	47 (24–94)	47 (24–94)	38 (27–69)	0.004
PASAT median score (range)	51 (11–60)	52 (12–60)	49 (11–59)	0.09
Disease-modifying treatment (*n*)				
No therapy	51	38	13	0.40
Azathioprin	5	4	1	
Interferon	87	67	20	
Copaxone	19	16	3	
Mitoxantron	1	0	1	

Abbreviations: *DH* dominant hand, *EDSS* expanded disability status scale, *9 HPT* nine-hole peg test, *NDH* non-dominant hand, *PASAT* Paced Auditory Serial Addition Test, *RRMS* relapsing remitting MS, *SDMT* symbol digit modalities test, *SPMS* secondary progressive MS, *T25FWT* timed 25-foot walk test

**Table 2:** Baseline MRI volumes and annualized volume loss rates

	Total	RRMS	SPMS	*p*-level
	*n* = 163	*n* = 125	*n* = 38	
Total cerebral volume [cm3]				
Mean ± SD	1312 ± 94	1329 ± 90	1259 ± 90	< 0.001
Range	1055–1499	1058–1499	1055–1437	
Annual cerebral volume rate (%/y)				
Mean ± SD	− 0.5 ± 1.1	− 0.46 ± 1.05	− 0.62 ± 1.18	n.s
Range	− 6.2–1.6	− 6.24–1.59	− 4.96–1.48	
Supratentorial LV [mm3] range				
Median	3363	3290	3563	n.s
Range	0–30581	0–27136	14.3–30581	
Infratentorial LV [mm3] range				
Median	28.6	17.1	58.7	n.s
Range	0–2037	0–2037	0–826	
TCV [cm3]				
Mean ± SD	177 ± 19	180 ± 19	168 ± 19	< 0.001
Range	124–227	130 ± 227	124–209	
Annual TCV rate (%/y)				
Mean ± SD	− 0.43 ± 0.7	− 0.40 ± 0.70	− 0.53 ± 0.71	n.s
Range	− 2.9–1.6	− 2.87–1.63	− 2.36–1.58	
Cerebellar WM volume [cm3]				
Mean ± SD	26 ± 3.9	27 ± 4	25 ± 4	0.005
Range	15–36	17–36	15–31	
Annual cerebellar WM volume rate (%/y)				
Mean ± SD	− 0.22 ± 1.92	− 0.43 ± 1.43	0.39 ± 2.88	0.028
Range	− 4.46–12.56	− 4.46–2.96	− 3.52–12.56	
Cerebellar GM volume [cm3]				
Mean ± SD	151 ± 16	153 ± 16	143 ± 16	< 0.001
Range	108–191	113–191	108–179	
Annual cerebellar GM volume rate (%/y)				
Mean ± SD	− 0.46 ± 0.69	− 0.39 ± 0.69	− 0.67 ± 0.64	0.027
Range	− 3.1–1.4	− 3.07–1.43	− 2.69–0.25	
Ant. lobe volume [cm3]				
Mean ± SD	19 ± 2.5	19 ± 2.4	18 ± 2.7	0.007
Range	13–25	13–25	13–23	
Annual ant. lobe volume rate (%/y)				
Mean ± SD	− 0.62 ± 1.00	− 0.46 ± 0.69	− 0.78 ± 1.15	n.s
Range	− 5.49–1.60	4.61–1.60	− 5.49–0.68	
Post. sup. lobe volume [cm3]				
Mean ± SD	59 ± 7.6	60 ± 7.2	55 ± 7.7	< 0.001
Range	36–76	43–74	36–75	
Annual post. sup. lobe volume rate (%/y)				
Mean ± SD	− 0.43 ± 0.78	− 0.29 ± 0.72	− 0.82 ± 0.83	< 0.001
Range	− 3.96–2.68	− 3.96–2.68	− 3.34–0.82	
Post. inf. lobe volume [cm3]				
Mean ± SD	73 ± 8.2	74 ± 8.3	70 ± 7.2	0.008
Range	54–95	54–95	58–84	
Annual post. inf. lobe volume rate (%/y)				
Mean ± SD	− 0.45 ± 0.78	− 0.43 ± 0.79	− 0.53 ± 0.74	n.s
Range	− 3.61–1.59	− 3.61–1.59	− 2.72—0.73	

Abbreviations: *Ant*. anterior, *GM* grey matter, *inf*. inferior, *LV* lesion volume, *post*. posterior, *RRMS* relapsing remitting MS, *SD* standard deviation, *SPMS* secondary progressive MS, *sup*. superior, *TCV* total cerebellar volume, *WM* white matter

### Procedures

All patients received comprehensive assessment annually. This included a standardized neurological examination with Expanded Disability Status Scale (EDSS) by certified neurologists, timed 25-foot walk test (T25FWT), nine-hole peg test (9HPT) with the dominant and non-dominant hand, Paced Auditory Serial Addition Test (PASAT) and Symbol Digit Modality Test (SDMT).

All MRI scans were acquired on the same 1.5 T MR scanner (Magnetom Avanto, Siemens Healthineers, Erlangen, Germany). Morphological analyses were performed on 3D T1-weighted (T1w) magnetization-prepared rapid gradient-echo (MPRAGE) brain MRI scans acquired in sagittal orientation (TR/TI/TE = 2080/1100/3.0 ms; α = 15°, 160 slices, resolution: 0.98 × 0.98 × 1 mm^3^). Additionally, a double spin echo proton density (PD)/T2-weighted sequence was applied (TR/TE1/TE2 = 3980/14/108 ms; 40 slices 3 mm thick without gap with an in-plane resolution of 1 mm^2^). Each 3D T1w data set underwent automatic cerebellar and whole-brain segmentation. Total and regional cerebellar volumes were computed using the automated pipeline RASCAL^18^. The original pipeline was modified using an updated version of the MNI152 reference template with 30 mm larger coverage inferiorly and additionally a study-specific registration template created from 37 scans of the RASCAL library to improve non-linear registration around the cerebellum and brain stem [20]. For each data set, all individual cerebellar lobules, cerebellar peduncles and white matter core of both hemispheres were assessed. Based on the latter, the following volumes were generated: total cerebellar volume (TCV) as the sum of all lobules plus white matter core incl. peduncles; total cerebellar grey matter (CGV) as the sum of anterior lobes (I–V), posterior superior lobes (VI + Crus I) and posterior inferior lobes (Crus II-lobule X) of both hemispheres; and total cerebellar white matter (CWV) as the sum of cerebellar peduncles and white matter core of both hemispheres, including the deep cerebellar nuclei. Cerebellar volumes were normalized for head size and reported in the MNI152 stereotaxic space. Segmentations were visually inspected for quality and excluded from further statistical analysis in case of segmentation errors. White matter lesions were segmented on the PD/T2w images by trained expert MRI readers according to standard operating procedures used at the local institution for the analysis of clinical period II and period III trial as described before [21]. Lesion volumes (LV) were calculated according to their anatomical location with respect to the tentorium (so-called supratentorial and infratentorial LV). A second processing pipeline was set up to compute whole-brain parenchymal volume using the 3D T1w datasets (for details [22]). The cerebral volume contains the remaining tissue after subtracting the brainstem volume and TCV from the whole-brain parenchymal volume (extracted using BEaST [23]).

### Statistical Analyses

All statistical analyses were performed using R Version 3.2.3 (https://www.r-project.org/).

The mean annual volume loss rate of each structure was calculated for every patient as the average of the annualized changes between all available time points^1^. In order to approximate a normal distribution, a logarithmic transformation for the EDSS and 9HPT, an inverse transformation for the T25FWT and cubic transformation for the PASAT were conducted. Further statistical analyses were performed using the transformed clinical scores.

Between-group comparisons of baseline demographic factors, clinical measurements and number of follow-ups were performed using the following tests (where appropriate): Welch’s *t*-test, Pearson’s chi-squared test with Yate’s continuity correction and Mann–Whitney *U* tests. Between-group differences for baseline MRI measures and annual rates of change were performed using analyses of covariance (ANCOVA), corrected for age, sex and disease duration.

Linear mixed-effects regression (LMER) analyses were deployed to explore the longitudinal evolution of cerebellar and cerebral volumes as well as the associations between MRI changes and clinical measures (period I data). Analyses were conducted in the whole cohort and for subgroups separately. For simplification, we report only the subgroup analyses within the “Results” section. Results for the whole cohort are reported within the supplementary material only. LMER models were performed in a forward stepwise fashion, using a “random intercept” and a “random slope” to allow for within-subject and between-subject variance. Each factor was tested both for its contribution to the fit’s intercept as well as to the fit’s slope. The fit’s intercept corresponds to the average of the dependent variable, whereas the fit’s slope to the change of the dependent variable over time. Independent variables were entered blockwise keeping the following sequence: first demographics and clinical factors, then cerebellar volumes and finally cerebral volume. All independent variables without statistical significance were excluded from the final model.

In a second step, the predictive capabilities of the cross-sectional cerebellar volumes of the last available MRI (baseline of period II) and the annualized cerebellar volume changes (MRI data from period I) regarding clinical disease progression in the future (clinical data from period II) were analysed with LMER models as described above with baseline volumes added before annualized changes in the model.

All results were corrected for multiple comparisons using the false discovery rate approach set at *q* < 0.05.

Cerebellar volumes included in the analyses were as follows: TCV, CGV, CWV, anterior lobes, posterior superior and inferior lobes in case of all clinical parameters tested. Further, selected individual lobes depending on the probability to be involved in the processing of the included clinical test (hypothesis driven) were included in the analyses mentioned above, namely lobules I–IV in case of analyses of the T25FWT [24, 25]; sum of lobules IV–VI + VIII in case of analyses of the 9HPT of the dominant (DH9HPT) and non-dominant hand (NDH9HPT) [24, 25] and peduncle volumes in case of analyses of the PASAT and SDMT [12, 26, 27]. In the analyses including the 9HPT as the dependent variable, only the ipsilateral volumes were considered. For simplification we still refer to the volumes as named above.

## Results

Data of 125 pwRRMS and 38 pwSPMS were included in the analyses. All patients had a median follow-up of 4 years in period I (clinical visit and MRI) with a subgroup (*n* = 77) having a median clinical long-term follow-up of 6 additional years (period II). All cerebellar lobes showed volume reduction over time in both groups, with the highest atrophy rate in the anterior lobes. Detailed baseline data are reported in Tables 1 and 2.

### Cerebellar Volume Changes — Period I Analyses

**Fig. 1:**
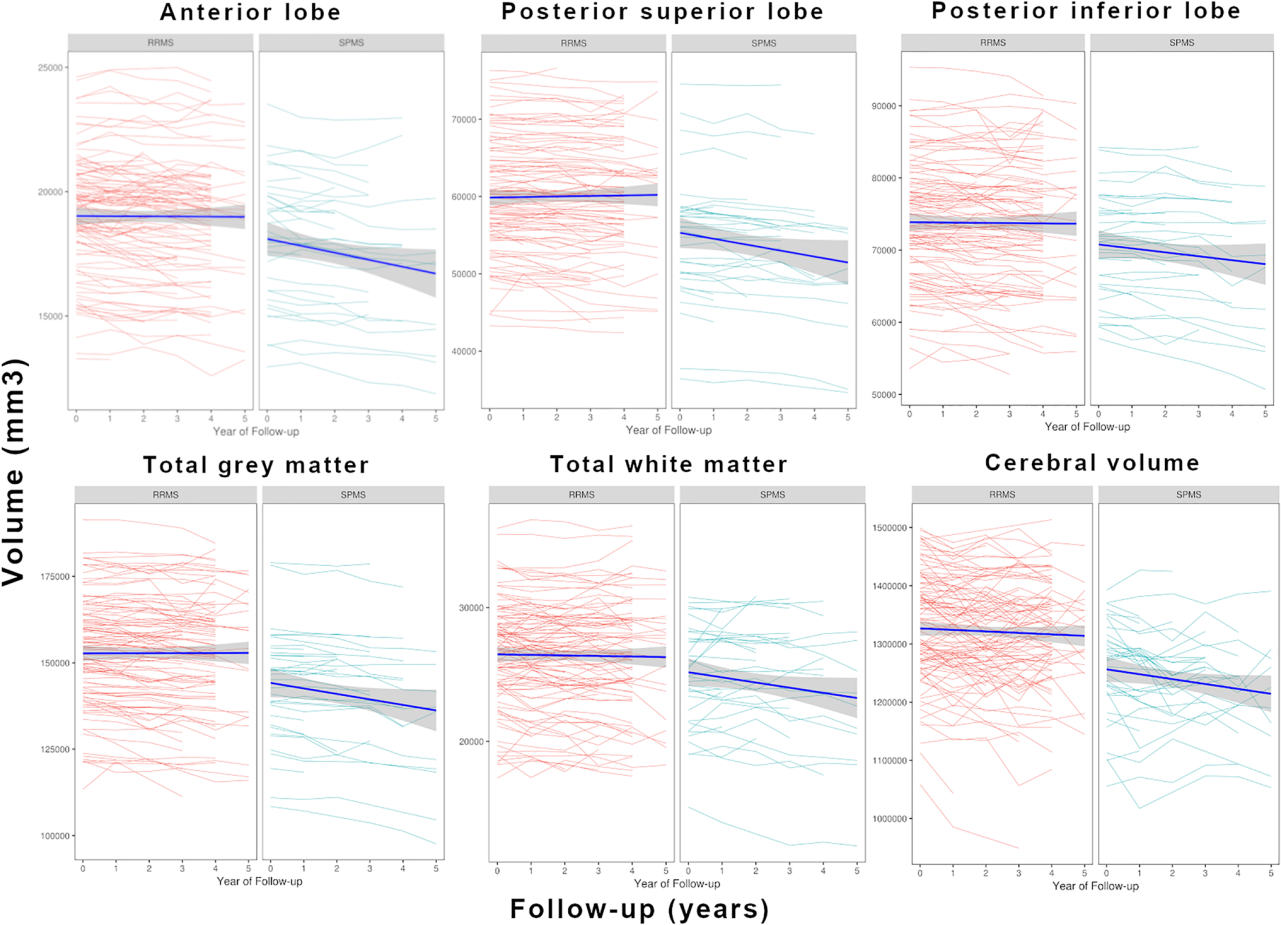
Upper row: individual volume trajectories over the follow-up years of RRMS (red) and SPMS (blue) of anterior lobes, posterior superior lobes and posterior inferior lobes. Lower row: individual volume trajectories over the follow-up years of RRMS (red) and SPMS (blue) of total grey matter and white matter as well as the cerebral volume for comparison. The thick blue lines represent the volume trajectories over the observation period for each group; confidence intervals are shown in grey

Cerebellar volumes (TCV, CGV, CWV, anterior lobe, posterior superior lobe, posterior inferior lobe — see Fig. 1) and their change over time were evaluated with respect to demographic and clinical metrics using LMER (Table 3). Analyses revealed that men, in general, had lower cerebellar volumes compared to women. In addition, in men, volume loss progressed faster compared to women in TCV, CGV, posterior superior lobe and posterior inferior lobe volume. Older age was associated with reduced cerebellar volumes on average and with faster volume loss over time for all cerebellar structures except for the anterior lobe. Disease duration was not associated with average cerebellar volumes or its loss over time. Further, disease subtype was not associated with cerebellar volumes on average, but pwSPMS exhibited faster posterior superior lobe volume loss over time compared to pwRRMS.

**Table 3:** Cerebellar volumes and their change over time with respect to demographic factors

	TCV (mm^3^)		CGV (mm^3^)		CWV (mm^3^)		Ant. lobe volume (mm^3^)		Post. sup. lobe volume (mm^3^)		Post. inf. lobe volume (mm^3^)	
	Average	Change over time	Average	Change over time	Average	Change over time	Average	Change over time	Average	Change over time	Average	Change over time
Sex (males)	− 13418 ± 2.774,***	− 408.7 ± 174.0,*	− 10639 ± 2334,***	− 370.2 ± 139.2,**	− 2781 ± 586.5,***	-	− 1228 ± 376.0,**	-	− 4987 ± 1097,***	− 111.2 ± 49.83,*	− 4420 ± 1261,**	− 201.1 ± 79.91,*
Age (years)	− 654.0 ± 114.9,***	− 21.49 ± 7.34,**	− 574.5 ± 96.68,***	− 15.36 ± 5.87,**	− 79.51 ± 24.33,**	− 6.27 ± 2.54,*	− 75.56 ± 15.58,**	-	− 277.2 ± 45.43,***	− 2.72 ± 2.22,*	− 220.7 ± 52.22,***	− 9.64 ± 3.36,**
DD (years)	-	-	-	-	-	-	-	-	-	-	-	-
MS subtype (SPMS)	-	-	-	-	-	-	-	-	-	− 126.4 ± 58.85,*	-	-
	Final model: R^2^m = 26%, R^2^c = 99%		Final model: R^2^m = 26%, R^2^c = 99%		Final model: R^2^m = 17%, R^2^c = 98%		Final model: R^2^m = 16%, R^2^c = 99%		Final model: R^2^m = 27%, R^2^c = 99%		Final model: R2m = 17%, R2c = 99%	

Abbreviations: *Ant*. anterior, *CGV* cerebellar grey matter volume, *CWV* cerebellar white matter volume, *inf*. inferior, *post*. posterior, *R*^*2*^_*m*_ marginal R-squared, *R*^*2*^_*c*_ conditional R-squared, *SPMS* secondary progressive MS, *sup*. superior, *TCV* total cerebellar volume

Significance level: *p* < 0.05 = *; *p* < 0.01 = **; *p* < 0.001 = ***

Analysis was performed with linear mixed-effect models with a random intercept and slope. Boxes display regression coefficients and respective levels of significance

Correcting for significant demographic factors, disease subtype and disease duration, we examined the association between cerebellar volumes, and infra- and supratentorial LV. Infratentorial LV were not associated with average cerebellar volumes or with cerebellar volume loss over time. Supratentorial LV was associated with all cerebellar volumes on average but the anterior lobe and the posterior superior lobe (TCV: B =  − 0.44 ± 0.14, *p* < 0.01; CGV: B =  − 0.28 ± 0.12, *p* < 0.05; CWV: B =  − 0.17 ± 0.03, *p* < 0.001; posterior inferior lobe: B =  − 0.17 ± 0.07, *p* < 0.05). Changes of supratentorial LV over time were also associated with changes of all cerebellar volumes over time but the anterior lobe (TCV: B =  − 0.04 ± 0.01, *p* < 0.01; CGV: B =  − 0.28 ± 0.12, *p* < 0.05; CWV: B =  − 0.17 ± 0.03, *p* < 0.001, posterior superior lobe: B =  − 0.01 ± 0.004, *p* < 0.01, posterior inferior lobe: B =  − 0.17 ± 0.07, *p* < 0.05).

We further examined the association between cerebellar volume and supratentorial cerebral volume, correcting for significant demographic factors, disease subtype and disease duration. Supratentorial cerebral volume was correlated with all cerebellar volumes on average (TCV: B = 0.007 ± 0.004, *p* < 0.01; CGV: B = 0.007 ± 0.003, *p* < 0.01; CWV: B = 0.003 ± 0.001, *p* < 0.01; anterior lobe: B = 0.002 ± 0.001, *p* < 0.001; posterior superior lobe: B = 0.001 ± 0.001, *p* < 0.05; posterior inferior lobe: B = 0.004 ± 0.002, *p* < 0.01). Changes of supratentorial cerebral volume were correlated with changes of all cerebellar volumes over time but CWV and anterior lobe (TCV: B = 0.002 ± 0.001, *p* < 0.05; CGV: B = 0.002 ± 0.001, *p* < 0.05; posterior superior lobe: B = 0.001 ± 0.0002, *p* < 0.001; posterior inferior lobe: B = 0.001 ± 0.0004, *p* < 0.05).

### Cerebellar Volume Changes and Disability — Period I Analyses

LMER in the pwRRMS subgroup (Table 4) showed that all cerebellar volumes were inversely correlated with the average EDSS, T25FWT and directly with the average SDMT. Average D9HPT and ND9HPT were inversely associated with the total volume of the cerebellar hemisphere and the sum of lobules IV, VI and VIII on the respective ipsilateral side. Peduncle and anterior lobe volumes were by trend correlated with the average PASAT. No correlation was found between changes of any clinical score over time and cerebellar volumes.

**Table 4:** Correlations between cerebellar volumes and clinical scores in RRMS patients

	log(EDSS)		1/T25FWT		log(D9HPT)		log(ND9HPT)		SDMT		PASAT^2^	
	Average	Change over time	Average	Change over time	Average	Change over time	Average	Change over time	Average	Change over time	Average	Change over time
TCV (mm^3^)	− 5 × 10^−6^ ± 2 × 10^−6^,**	-	10^−6^ ± 3 × 10^−7^,**	-	− 7 × 10^−6^ ± 2 × 10^−6^,*	-	− 7 × 10^−6^ ± 2 × 10^−6^,*	-	10^−4^ ± 5 × 10^−5^,**	-	-	-
Cerebrum (mm^3^)	-	-	-	-	-	-	-	-	2 × 10^−5^ ± 8 × 10^−6^,**	-	-	-
	Final model: R^2^m = 19%, R^2^c = 89%		Final model: R^2^m = 19%, R^2^c = 93%		Final model: R^2^m = 12%, R^2^c = 92%		Final model: R^2^m = 6%, R^2^c = 89%		Final model: R^2^m = 27%, R^2^c = 92%		-	
CGV (mm^3^)	− 5 × 10^−6^ ± 2 × 10^−6^,**	-	10^−6^ ± 3 × 10^−7^,**	-	-	-	-	-	10^−4^ ± 6 × 10^−5^,*	-	-	-
Cerebrum (mm^3^)	− 6 × 10^−7^ ± 3 × 10^−7^,*	-	-	-	-	-	-	-	2 × 10^−5^ ± 8 × 10^–6^,**	-	-	-
	Final model: R^2^m = 19%, R^2^c = 88%		Final model: R^2^m = 18%, R^2^c = 93%		-		-		Final model: R^2^m = 27%, R^2^c = 92%		-	
CWV (mm^3^)	− 2 × 10^−5^ ± 7 × 10^–6^,***	-	3 × 10^−6^ ± 10^−6^,**	-	-	-	-	-	7 × 10^−4^ ± 2 × 10^−4^,***	-	-	-
Cerebrum (mm^3^)	-	-	-	-	-	-	-	-	2 × 10^−5^ ± 8 × 10^−6^,*	-	-	-
	Final model: R^2^m = 19%, R^2^c = 89%		Final model: R^2^m = 16%, R^2^c = 93%		-		-		Final model: R^2^m = 28%, R^2^c = 92%		-	
Ant. lobe (mm^3^)	− 4.3 × 10^−5^ ± 10^−5^,**	-	6 × 10^−6^ ± 2 × 10^−6^,**	-	-	-	-	-	5 × 10^−4^ ± 4 × 10^−4^,*	-	0.07 ± 0.03, *q* = 0.060	-
Cerebrum (mm^3^)	-	-	-	-	-	-	-	-	3 × 10^−5^ ± 8 × 10^−6^,**	-	-	-
	Final model: R^2^m = 19%, R^2^c = 89%		Final model: R^2^m = 16%, R^2^c = 93%		-		-		Final model: R^2^m = 25%, R^2^c = 92%		Final model: R^2^m = 12%, R^2^c = 71%	
Post. sup. lobe (mm^3^)	-	-	10^−6^ ± 7 × 10^−7^,*	-	-	-	-	-	3 × 10^−4^ ± 10^−4^,*	-	-	-
Cerebrum (mm^3^)	-	-	7 × 10^–8^ ± 4 × 10^−8^,*	-	-	-	-	-	3 × 10^−5^ ± 8 × 10^−6^,**	-	-	-
	-		Final model: R^2^m = 16%, R^2^c = 93%		-		-		Final model: R^2^m = 26%, R^2^c = 92%		-	
Post. inf. lobe (mm^3^)	− 9 × 10^−6^ ± 3 × 10^−6^, **	-	2 × 10^−6^ ± 5 × 10^−7^,**	-	-	-	-	-	2 × 10^−4^ ± 10^−4^,*	-	-	-
Cerebrum (mm^3^)	− 6 × 10^−7^ ± 3 × 10^−7^, *	-	7 × 10^−8^ ± 4 × 10^−8^,*	-	-	-	-	-	3 × 10^−5^ ± 8 × 10^−6^,**	-	-	-
	Final model: R^2^m = 19%, R^2^c = 88%		Final model: R^2^m = 19%, R^2^c = 93%		-		-		Final model: R^2^m = 26%, R^2^c = 92%		-	
Lobules IV, VI and VIII (mm^3^)	n.e	n.e	n.e	n.e	− 2 × 10^−5^ ± 6 × 10^−6^,*	-	− 2 × 10^−5^ ± 6 × 10^−6^,*	-	n.e	n.e	n.e	n.e
Cerebrum (mm^3^)	n.e	n.e	n.e	n.e	-	-	-	-	n.e	n.e	n.e	n.e
	-		-		Final model: R^2^m = 10%, R^2^c = 92%		Final model: R^2^m = 3%, R^2^c = 89%		-		-	
Cerebellar Peduncles (mm^3^)	n.e	n.e	n.e	n.e	n.e	n.e	n.e	n.e	10^−3^ ± 4 × 10^−4^,***	-	0.08 ± 0.03, *q* = 0.078	-
Cerebrum (mm^3^)	n.e	n.e	n.e	n.e	n.e	n.e	n.e	n.e	2 × 10^−5^ ± 8 × 10^−6^,*	-	-	-
	-		-		-		-		Final model: R^2^m = 29%, R^2^c = 92%		Final model: R^2^m = 11%, R^2^c = 71%	

Abbreviations: *Ant*. = anterior, *Cerebrum* (supratentorial) cerebral volume, *CGV* cerebellar grey matter volume, *CWV* cerebellar white matter volume, *D9HPT* dominant-hand 9-hole peg test, *EDSS* Expanded Disability Status Scale, *inf*. inferior, *ND9HPT* non-dominant-hand 9-hole peg test, *n.e.* not evaluated, *PASAT* Paced Auditory Serial Addition Test, *Post*. posterior, *R*^*2*^_*m*_ marginal R-squared, *R*^*2*^_*c*_ conditional R-squared, *SDMT* Single Digit Modality Test, *T25FWT* timed 25-foot walk test, *TCV* total cerebellar volume

Significance level after correcting using the false discovery rate method: *q* < 0.05 = *, *q* < 0.01 = **, *q* < 0.001 = ***

Analysis was performed with linear mixed-effect models with a random intercept and slope. In all models, independent variables were entered blockwise keeping the following sequence: first demographics (sex, age) and clinical factors (disease duration), then cerebellar volumes and finally supratentorial cerebral volume. Only ipsilateral cerebellar volumes were tested in the analysis of D9HPT and ND9HPT. Each factor was tested both for its contribution to the fit’s intercept as well as to the fit’s slope. All independent variables without statistical significance were excluded from the final model. Boxes display regression coefficients and respective levels of significance

LMER in the pwSPMS subgroup showed a trend to an inverse correlation between posterior superior lobe volume changes and EDSS changes over time (B =  − 10^−6^ ± 4 × 10^−7^, *q* = 0.085; final model: R_2_m = 67%, R_2_c = 92%). Cerebral volume did not contribute further in this model. No other associations between cerebellar volumes and clinical scores were found.

### Cerebellar Volume Changes and Prediction of Future Disability — Period II Analyses

LMER in the pwRRMS subgroup (Table 5) showed that all baseline cerebellar volumes were inversely correlated with the future average T25FWT and all baseline cerebellar volumes but the posterior inferior lobe were inversely correlated with the future average EDSS and SDMT. Baseline cerebral volume also contributed in these models. However, no cerebellar metrics were associated with future EDSS, T25FWT and SDMT changes over time. Baseline TCV, baseline CGV, baseline posterior superior lobe volume (by trend) as well as the sum of cerebellar lobules IV, VI and VIII (baseline and by trend with annual changes) and annual CWV changes were associated with the average future D9HPT, whereas no cerebellar metrics were correlated with faster D9HPT worsening over time. Further, all baseline cerebellar volumes (but baseline CGV) as well as the annual volume changes of CGV, CWV, posterior inferior lobe and the summed cerebellar lobules IV, VI and VIII were associated with the average future ND9HPT, whereas higher annual atrophy of the summed volumes of cerebellar lobules IV, VI and VIII and by trend of the TCV were correlated to faster ND9HPT worsening over time. Cerebral volume also contributed in the 9HPT analyses. No cerebellar metrics were correlated with PASAT.

**Table 5:** Correlations between annual cerebellar volume change rates and future clinical scores in RRMS patients

MRI metrics	log(EDSS)		1/T25FWT		log(D9HPT)		log(ND9HPT)			SDMT		PASAT^2^	
	Average	Change over time	Average	Change over time	Average	Change over time	Average	Change over time		Average	Change over time	Average	Change over time
Baseline TCV (mm^3^)	− 3.8 × 10^−6^ ± 1.8 × 10^−6^,***	-	6.8 × 10^−7^ ± 3.1 × 10^−7^,**	-	− 1.9 × 10^−6^ ± 3.9 × 10^−6^,*		− 9.6 × 10^−6^ ± 2.7 × 10^−6^,**	-		1.1 × 10^−4^ ± 6.2 × 10^−5^,*	-	-	-
TCV AVCR (%)	-	-	-	-	-	-	-	1.1 × 10^−2^ ± 4.2 × 10^−3^, *q* = 0.055		-	-	-	-
Baseline cerebrum (mm^3^)	− 1.5 × 10^−6^ ± 3.7 × 10^−7^,***	-	2.1 × 10^−7^ ± 6.7 × 10^−8^,**	-	− 8.3 × 10^−7^ ± 3.3 × 10^−7^,**	-	-	-		2.7 × 10^−5^ ± 1.3 × 10^−5^,*	-	n.e	n.e
Cerebrum AVCR (%)	-	-	-	-	-		-	− 1.2 × 10^−2^ ± 3.8 × 10^−3^,*		-	-	n.e	n.e
	Final model: R^2^m = 34%, R^2^c = 86%		Final model: R^2^m = 33%, R^2^c = 90%		Final model: R^2^m = 35%, R^2^c = 94%		Final model: R^2^m = 34%, R^2^c = 92%			Final model: R^2^m = 30%, R^2^c = 91%		-	
Baseline CGV (mm^3^)	− 4.4 × 10^−6^ ± 2.1 × 10^−6^,***	-	8.3 × 10^−7^ ± 3.7 × 10^−7^,**	-	− 1.0 × 10^−5^ ± 1.0 × 10^−6^,*	-	-		-	1.3 × 10^−4^ ± 7.3 × 10^−5^,*	-	-	-
CGV AVCR (%)	-	-	-	-	-	-	− 0.11 ± 0.04,**		-	-	-	-	-
Baseline cerebrum (mm^3^)	− 1.6 × 10^−6^ ± 3.7 × 10^−7^,***	-	2.1 × 10^−7^ ± 6.7 × 10^−8^,**	-	-	-	-		-	2.8 × 10^−5^ ± 1.3 × 10^−5^,*	-	n.e	n.e
Cerebrum AVCR (%)	-	-	-	-	-	-	-		-	-	-	n.e	n.e
	Final model: R^2^m = 34%, R^2^c = 86%		Final model: R^2^m = 33%, R^2^c = 90%		Final model: R^2^m = 24%, R^2^c = 90%		Final model: R^2^m = 23%, R^2^c = 89%			Final model: R^2^m = 30%, R^2^c = 91%		-	
Baseline CWV (mm^3^)	− 1.6 × 10^−6^ ± 8.7 × 10^−6^,***	-	2.1 × 10^−6^ ± 1.4 × 10^−6^,**	-	-	-	− 2.0 × 10^−5^ ± 1.3 × 10^−5^,*		-	6.6 × 10^−4^ ± 2.8 × 10^−4^,*	-	-	-
CWV AVCR (%)	-	-	-	-	− 0.06 ± 0.02, *	-	− 0.04 ± 0.02,*		-	-	-	-	-
Baseline cerebrum (mm^3^)	− 1.6 × 10^−6^ ± 3.8 × 10^–7^,***	-	2.2 × 10^−7^ ± 6.9 × 10^−8^,**	-	-	-	-		-	-	-	n.e	n.e
Cerebrum AVCR (%)	-	-	-	-	-	-	-		-	-	-	n.e	n.e
	Final model: R^2^m = 33%, R^2^c = 86%		Final model: R^2^m = 31%, R^2^c = 90%		Final model: R^2^m = 22%, R^2^c = 90%		Final model: R^2^m = 25%, R^2^c = 89%			Final model: R^2^m = 27%, R^2^c = 91%		-	
Baseline ant. lobe (mm^3^)	− 4.0 × 10^–5^ ± 1.4 × 10^−5^,***	-	5.3 × 10^−6^ ± 2.4 × 10^−6^,**	-	-	-	− 5.4 × 10^−5^ ± 1.8 × 10^−5^,*		-	-	-	-	-
Ant. lobe AVCR (%)	-	-	-	-	-	-	-		-	-	-	-	-
Baseline cerebrum (mm^3^)	− 1.5 × 10^−6^ ± 3.6 × 10^−7^,***	-	2.1 × 10^−7^ ± 6.6 × 10^−8^,**	-	n.e	n.e	-		-	n.e	n.e	n.e	n.e
Cerebrum AVCR (%)	-	-	-	-	n.e	n.e	-		-	n.e	n.e	n.e	n.e
	Final model: R^2^m = 36%, R^2^c = 86%		Final model: R^2^m = 33%, R^2^c = 90%		-		Final model: R^2^m = 24%, R^2^c = 89%			-		-	
Baseline post. sup. lobe (mm^3^)	− 3.4 × 10^−6^ ± 4.7 × 10^−6^,***	-	1.1 × 10^−6^ ± 8.1 × 10^−7^,*	-	− 1.9 × 10^−5^ ± 8.2 × 10^−6^, *q* = 0.051	-	− 2.5 × 10^−5^ ± 6.3 × 10^−6^,***		-	3.9 × 10^−4^ ± 1.6 × 10^−4^,*	-	-	-
Post. sup. lobe AVCR (%)	-	-	-	-	-	-	-		-	-	-	-	-
Baseline cerebrum (mm^3^)	− 1.8 × 10^−6^ ± 3.7 × 10^−7^,***	-	2.3 × 10^−7^ ± 6.7 × 10^−8^,***	-	-	-	-		-	2.7 × 10^−5^ ± 1.3 × 10^−5^,*	-	n.e	n.e
Cerebrum AVCR (%)	-	-	-	-	-	-	-		-	-	-	n.e	n.e
	Final model: R^2^m = 31%, R^2^c = 86%		Final model: R^2^m = 31%, R^2^c = 90%		Final model: R^2^m = 22%, R^2^c = 90%		Final model: R^2^m = 28%, R^2^c = 89%			Final model: R^2^m = 30%, R^2^c = 91%		-	
Baseline post. inf. lobe (mm^3^)	-	-	1.5 × 10^−6^ ± 6.8 × 10^−7^,**	-	-		− 7.8 × 10^−5^ ± 5.7 × 10^−6^,*		-	-	-	-	-
Post. inf. lobe AVCR (%)	-	-	-	-	-	-	− 9.4 × 10^−2^ ± 3.2 × 10^−2^,*		-	-	-	-	-
Baseline cerebrum (mm^3^)	n.e	n.e	2.2 × 10^−7^ ± 6.5 × 10^−8^,**	-	n.e	n.e	-		-	n.e	n.e	n.e	n.e
Cerebrum AVCR (%)	n.e	n.e	-	-	n.e	n.e	-		-	n.e	n.e	n.e	n.e
	-		Final model: R^2^m = 33%, R^2^c = 90%		-		Final model: R^2^m = 25%, R^2^c = 89%			-		-	
Baseline lobules IV, VI and VIII (mm^3^)	n.e	n.e	n.e	n.e	− 1.7 × 10^−5^ ± 8.5 × 10^−6^,*	-	− 2.5 × 10^−5^ ± 7.1 × 10^−6^,***		-	n.e	n.e	n.e	n.e
Lobules IV, VI and VIII AVCR (%)	n.e	n.e	n.e	n.e	− 0.03 ± 0.03, *q* = 0.066		− 6.4 × 10^−3^ ± 2.3 × 10^−2^,*		0.011 ± 0.004,**	n.e	n.e	n.e	n.e
Baseline cerebrum (mm^3^)	n.e	n.e	n.e	n.e	− 1.7 × 10^−5^ ± 8.5 × 10^–6^,**	-	− 5.2 × 10^−7^ ± 2.6 × 10^−7^,*		-	n.e	n.e	n.e	n.e
Cerebrum AVCR (%)	n.e	n.e	n.e	n.e	-	− 1.4 × 10^−3^ ± 5.3 × 10^−3^,*	-		− 8.2 × 10^−3^ ± 4.0 × 10^−3^,*	n.e	n.e	n.e	n.e
	-		-		Final model: R^2^m = 33%, R^2^c = 94%		Final model: R^2^m = 34%, R^2^c = 92%			-		-	
Baseline peduncles (mm^3^)	n.e	n.e	n.e	n.e	n.e	n.e	n.e		n.e	1.8 × 10^−3^ ± 5.7 × 10^−4^,*	-	-	-
Peduncles AVCR (%)	n.e	n.e	n.e	n.e	n.e	n.e	n.e		n.e	-	-	-	-
Baseline cerebrum (mm^3^)	n.e	n.e	n.e	n.e	n.e	n.e	n.e		n.e	-	-	n.e	n.e
Cerebrum AVCR (%)	n.e	n.e	n.e	n.e	n.e	n.e	n.e		n.e	-	-	n.e	n.e
	-		-		-		-			Final model: R^2^m = 29%, R^2^c = 91%		-	

Abbreviations: *AVCR* annual volume change rate, *Ant*. anterior, *Cerebrum* (supratentorial) cerebral volume, *CGV* cerebellar grey matter volume, *CWV* cerebellar white matter volume, *D9HPT* dominant-hand 9-hole peg test, *EDSS* Expanded Disability Status Scale, *inf*. inferior, *ND9HPT* non-dominant-hand 9-hole peg test, *n.e*. not evaluated, *PASAT* Paced Auditory Serial Addition Test, *Post*. posterior, *RRMS* relapsing–remitting MS, *R*^*2*^_*m*_ marginal R-squared, *R*^*2*^_*c*_ conditional R-squared, *SDMT* Single Digit Modality Test, *sup*. superior, *T25fwt* timed 25-foot walk test, *TCV* total cerebellar volume

Significance level after correcting using the false discovery rate method: *q* < 0.05 = *, *q* < 0.01 = **, *q* < 0.001 = ***

Analysis was performed with linear mixed-effect models with a random intercept and slope. In all models, independent variables were entered blockwise keeping the following sequence: first demographics (sex, age) and clinical factors (disease duration), then cerebellar volumes and finally supratentorial cerebellar volume. Only ipsilateral cerebellar volumes were tested in the analysis of D9HPT and ND9HPT. Each factor was tested both for its contribution to the fit’s intercept as well as to the fit’s slope. All independent variables without statistical significance were excluded from the final model. Boxes display regression coefficients and respective levels of significance

LMER in the pwSPMS subgroup (Table 6) showed that the annual volume change rate of the anterior lobe was positively associated with changes of future ND9HPT over time. Average future PASAT was correlated with annual change rates of CGV, anterior lobe and posterior superior lobe volume, whereas annual volume change rates in the posterior superior lobe were correlated with future PASAT changes over time. No other associations between cerebellar volumes and clinical scores were found.

**Table 6:** Correlations between annual cerebellar volume change rates and future clinical scores in SPMS patients

MRI metrics	log(EDSS)		1/T25FWT		log(D9HPT)		log(ND9HPT)		SDMT		PASAT^2^	
	Average	Change over time	Average	Change over time	Average	Change over time	Average	Change over time	Average	Change over time	Average	Change over time
Baseline TCV (mm^3^)	-	-	-	-	-	-	-	-	-	-	-	-
TCV AVCR (%)	-	-	-	-	-	-	-	-	-	-	-	-
Baseline cerebrum (mm^3^)	n.e	n.e	n.e	n.e	n.e	n.e	n.e	n.e	n.e	n.e	n.e	n.e
Cerebrum AVCR (%)	n.e	n.e	n.e	n.e	n.e	n.e	n.e	n.e	n.e	n.e	n.e	n.e
	-		-		-		-		-		-	
Baseline CGV (mm^3^)	-	-	-	-	-	-	-	-	-	-	-	-
CGV AVCR (%)	-	-	-	-	-	-	-	-	-	-	666.0 ± 246.2,*	-
Baseline cerebrum (mm^3^)	n.e	n.e	n.e	n.e	n.e	n.e	n.e	n.e	n.e	n.e	n.e	n.e
Cerebrum AVCR (%)	n.e	n.e	n.e	n.e	n.e	n.e	n.e	n.e	n.e	n.e	n.e	n.e
	-		-		-		-		-		Final model: R^2^m = 10%, R^2^c = 57%	
Baseline CWV (mm^3^)	-	-	-	-	-	-	-	-	-	-	-	-
CWV AVCR (%)	-	-	-	-	-	-	-	-	-	-	-	-
Baseline cerebrum (mm^3^)	n.e	n.e	n.e	n.e	n.e	n.e	n.e	n.e	n.e	n.e	n.e	n.e
Cerebrum AVCR (%)	n.e	n.e	n.e	n.e	n.e	n.e	n.e	n.e	n.e	n.e	n.e	n.e
	-		-		-		-		-		-	
Baseline ant. lobe (mm^3^)	-	-	-	-	-	-	-	-	-	-	-	-
Ant. lobe AVCR (%)	-	-	-	-	-	-	-	0.063 ± 0.21,*	-	-	352.6 ± 119.8,*	-
Baseline cerebrum (mm^3^)	n.e	n.e	n.e	n.e	n.e	n.e	-	-	n.e	n.e	n.e	n.e
Cerebrum AVCR (%)	n.e	n.e	n.e	n.e	n.e	n.e	-	-	n.e	n.e	n.e	n.e
	-		-		-		Final model: R^2^m = 24%, R^2^c = 97%		-		Final model: R^2^m = 8%, R^2^c = 62%	
Baseline post. sup. lobe (mm^3^)	-	-	-	-	-	-	-	-	-	-	-	-
Post. sup. lobe AVCR (%)	-	-	-	-	-	-	-	-	-	-	− 248.6 ± 296.6,*	302.9 ± 72.8,**
Baseline cerebrum (mm^3^)	n.e	n.e	n.e	n.e	n.e	n.e	n.e	n.e	n.e	n.e	n.e	n.e
Cerebrum AVCR (%)	n.e	n.e	n.e	n.e	n.e	n.e	n.e	n.e	n.e	n.e	n.e	n.e
	-		-		-		-		-		Final model: R^2^m = 41%, R^2^c = 61%	
Baseline post. inf. lobe (mm^3^)	-	-	-	-	-	-	-	-	-	-	-	-
Post. inf. lobe AVCR (%)	-	-	-	-	-	-	-	-	-	-	-	-
Baseline cerebrum (mm^3^)	n.e	n.e	n.e	n.e	n.e	n.e	n.e	n.e	n.e	n.e	n.e	n.e
Cerebrum AVCR (%)	n.e	n.e	n.e	n.e	n.e	n.e	n.e	n.e	n.e	n.e	n.e	n.e
	-		-		-		-		-		-	
Baseline lobules IV, VI and VIII (mm^3^)	n.e	n.e	n.e	n.e	-	-	-	-	n.e	n.e	n.e	n.e
Lobules IV, VI and VIII AVCR (%)	n.e	n.e	n.e	n.e			-	-	n.e	n.e	n.e	n.e
Baseline cerebrum (mm^3^)	n.e	n.e	n.e	n.e	n.e	n.e	n.e	n.e	n.e	n.e	n.e	n.e
Cerebrum AVCR (%)	n.e	n.e	n.e	n.e	n.e	n.e	n.e	n.e	n.e	n.e	n.e	n.e
	-		-		n.e		-		-		-	
Baseline peduncles (mm^3^)	n.e	n.e	n.e	n.e	n.e	n.e	n.e	n.e	-	-	-	-
Peduncles AVCR (%)	n.e	n.e	n.e	n.e	n.e	n.e	n.e	n.e	-	-	-	-
Baseline cerebrum (mm^3^)	n.e	n.e	n.e	n.e	n.e	n.e	n.e	n.e	n.e	n.e	n.e	n.e
Cerebrum AVCR (%)	n.e	n.e	n.e	n.e	n.e	n.e	n.e	n.e	n.e	n.e	n.e	n.e
	-		-		-		-		-		-	

Abbreviations: *AVCR* annual volume change rate, *Ant*. anterior, *Cerebrum* (supratentorial) cerebral volume, *CGV* cerebellar grey matter volume, *CWV* cerebellar white matter volume, *D9HPT* dominant-hand 9-hole peg test, *EDSS* Expanded Disability Status Scale, *inf*. inferior, *ND9HPT* non-dominant-hand 9-hole peg test, *n.e*. not evaluated, *PASAT* Paced Auditory Serial Addition Test, *Post*. posterior, *RRMS* relapsing–remitting MS, *R*^*2*^_*m*_ marginal R-squared, *R*^*2*^_*c*_ conditional R-squared, *SDMT* Single Digit Modality Test, *sup*. superior, *T25fwt* timed 25-foot walk test, *TCV* total cerebellar volume

Significance level after correcting using the false discovery rate method: *q* < 0.05 = *, *q* < 0.01 = **, *q* < 0.001 = ***

Analysis was performed with linear mixed-effect models with a random intercept and slope. In all models, independent variables were entered blockwise keeping the following sequence: first demographics (sex, age) and clinical factors (disease duration), then cerebellar volumes and finally supratentorial cerebellar volume. Only ipsilateral cerebellar volumes were tested in the analysis of D9HPT and ND9HPT. Each factor was tested both for its contribution to the fit’s intercept as well as to the fit’s slope. All independent variables without statistical significance were excluded from the final model. Boxes display regression coefficients and respective levels of significance

## Discussion

Previous studies have shown a relation between reduced cerebellar volumes in pwMS and sensory-motor dysfunction as well as cognitive-behavioural tasks [13, 17, 22, 28]; however, little is known on the temporal evolution of cerebellar volume loss and the corresponding deterioration of function. In this work, we analysed a large cohort over a long clinical observation period of up to 11 years. We could show significant volume reduction in the cerebellum and its substructures in relapse-onset MS patients and their relationship between regional cerebellar volume loss and motor and cognitive function over time.

In our cohort, pwSPMS exhibited faster posterior superior lobe volume loss over time compared to pwRRMS, a region known to be activated during various language, working memory and executive function tasks [29]. This is in line with earlier cross-sectional studies describing predominant cerebellar volume reduction in progressive patients over relapsing and benign MS forms [9, 15]. Average infratentorial LV or its change over time was neither associated with average cerebellar volume nor its changes over time, indicating at least partial independence between the formation of regional lesional burden (primarily inflammatory processes) and neurodegenerative processes leading to atrophy. The lack of association may on the other hand be explained by technical challenges in infratentorial space leading to limited lesion detection especially cortical lesions. In contrast, cerebral and cerebellar volumes correlated significantly. There is no preferential volume loss of cerebral over cerebellar tissue, as described before [30].

As to the clinical consequences of cerebellar volume reduction, we could confirm previous data in finding significant relations between average cerebellar volumes on a global as well as on a defined regional level and clinical test performance [10, 13, 17, 22]. However, for pwRRMS no associations were found when analysing the relation between regional volume loss over time and changes in the corresponding task performance over time. In contrast, for pwSPMS volume reduction in the posterior superior lobe (which was discriminative between both groups) over the study period was at least by trend related to increase of EDSS over time. While EDSS represents a measure for predominantly motor disability, the posterior superior lobe (including lobule VI and Crus I) — as mentioned before — has been predominantly related with higher order cognitive tasks (e.g. language, spatial tasks, executive function and affective processing). However, some studies have also shown involvement of this region with motor tasks and suggest relations of the posterior cerebellar regions with higher order motor planning [24, 26, 29, 31–33]. These and our findings suggest that not only direct damage of motor pathways may influence EDSS changes, and that neurodegeneration within associative centres affects motor performance in an indirect fashion. In addition, pwSPMS with longer disease duration and older age in general may have reduced adaptive and repair mechanisms to make up for loss of neuronal tissue, which again may translate more directly into dysfunction than in pwRRMS [34, 35].

Additionally, we were interested in determining whether cerebellar structural changes may predict future clinical disease progression. In pwRRMS, atrophy rates of cerebellar lobes and total volumes together with supratentorial cerebral volume were significant predictors of disease severity in terms of motor scores (e.g. average EDSS, T25FWT, 9HPT of both hands) and the SDMT, confirming previous results of cross-sectional analyses [13, 17, 22]. No relation was found between atrophy rates and future cognitive decline in this patient group, which may be partially explained by a more prominent learning effect for both tests (SDMT: 0.459 ± 0.667/year; PASAT^2^: 14.35 ± 18.65/year) as seen in other studies before [36, 37]. Another explanation may be the concept of “brain and cognitive reserve”, where cognitive performance may not correlate with brain atrophy due to “protective effects” of maximal life time brain volume (genetics) and premorbid intelligence and educational levels (environmental factors) [38].

Interestingly, we showed that lower baseline volumes and higher atrophy rates of ipsilateral hemispherical structures and in particular motor-associated lobules were significant predictors of faster future performance worsening on the 9HPT of the non-dominant hand in all pwMS. The cerebellum, in particular its anterior and superior posterior lobe, is activated as part of a large visuo-motor network including also fronto-parieto-occipital regions when dexterity tasks are performed by healthy controls. It shows even stronger activations when using the non-dominant over the dominant hand [32]. The 9HPT of the non-dominant hand further seems to better reflect real life upper limb disability [39] and may therefore be a more sensitive measure (than e.g. the EDSS) to volume changes even in less affected patients like RRMS. In pwSPMS, we showed that atrophy rates of the posterior superior lobe were a significant predictor of future PASAT performance deterioration. PASAT performance is known as a measure of processing speed, working memory and attention, activating a broad bilateral mainly fronto-parietal network but also including subcortical structures and the anterior and posterior superior cerebellum [40, 41]. Our results underline the importance of the cerebellar structures within this network, where neurodegeneration leads to faster deterioration of processing speed and attention.

General findings in this study were lower head-sized normalized cerebellar volumes in men than women, as previously described [18]. Further, men showed higher rates of volume loss over time especially within the posterior lobe grey matter, an area (amongst other functions) well-known to be part of cognitive-behavioural processing^3^. This might support the view of male sex being a risk factor for faster disability accumulation in relapse-onset patients [42]. Further, older age was associated with lower volumes and faster volume loss, but disease duration did not seem to be a relevant factor. Unfortunately, for this study no healthy matched control group was available, which could help in estimating the present cerebellar volume reduction in relation to healthy ageing. However, in general, the association between cerebellar volume loss and disability is not affected by this limitation.

The retrospective, longitudinal design is prone to potential bias due to dropout of patients. PwSPMS were smaller in number; however, they showed similar follow-up time than pwRRMS and therefor dropout is probably not due to disease progression. About 69% of patients were treated with disease-modifying therapy with dominance of first-line injectables, which potentially may bias volume analyses. This effect should, however, be negligible for the latter therapeutics [43]. Another limitation is the lack of data on atrophy of the deep cerebellar nuclei, which are an important relay station for motor and cognitive pathways from and to the cerebellum and might better reflect disability progression. Unfortunately, the quality of the T1w MRI data did not allow a reliable segmentation of the latter. High-field MRI data would be of advantage to look into the possible specific predictive value of the cerebellar nuclei for future disease progression.

## Conclusion

Overall, we conclude that the cerebellum not only plays an important role for motor and cognitive function in MS, but also reflects decline in clinical motor and cognitive performance and may even serve as a predictor for future disability, especially when dexterity and processing speed performance is in focus.

## Supplementary Information

Below is the link to the electronic supplementary material.

Supplementary file1 (DOCX 59 KB)

## Data Availability

The data that support the findings of this study are available on request from the corresponding author. The data are not publicly available due to privacy or ethical restrictions.
