# Supplementary material for: Regional Cerebellar Volume Loss Predicts Future Disability in Multiple Sclerosis Patients
Source: Cerebellum. 2021 Aug 21;21(4):632–46. doi: 10.1007/s12311-021-01312-0 (PMC9325849; doi:10.1007/s12311-021-01312-0)
Supplement: Supplementary file 1 — Supplementary file1 (DOCX 59 KB) [file 12311_2021_1312_MOESM1_ESM.docx]

**Supplementary material**

**Results of the analyses of the total cohort**

**Cerebellar volume changes and disability – phase I analyses**

Linear mixed effects regression (LMER) analyses in the whole cohort (Table SM1) showed that all cerebellar volumes except the posterior superior lobe were inversely correlated with the average EDSS and T25FWT. Average D9HPT was by trend inversely associated with the TCV. In addition, all cerebellar volumes but anterior and posterior inferior lobe were also associated with the average SDMT. No correlation was found between changes of any clinical score over time and cerebellar volumes.

**Table SM1. Correlations between cerebellar volumes and clinical scores in all relapse onset MS patients.**

|  | **log(EDSS)** | | **1/T25FWT** | | **log(D9HPT)** | | **log(ND9HPT)** | | **SDMT** | | **PASAT^2^** | |
| --- | --- | --- | --- | --- | --- | --- | --- | --- | --- | --- | --- | --- |
|  | **Average** | **Change over time** | **Average** | **Change over time** | **Average** | **Change over time** | **Average** | **Change over time** | **Average** | **Change over time** | **Average** | **Change over time** |
| **TCV (mm^3^)** | -4 x 10^-6^ ± 10^-6^,* | - | 7 x 10^-7^ ± 2 x 10^-7^,** | - | -6 x 10-6 ± 2 x 10-6, q=0.86 | - | - | - | 10-4 ± 5 x 10-5,** | - | - | - |
| **Cerebrum (mm^3^)** |  | - | - | - | - | - | - | - | 2 x 10-5 ± 7 x 10-6,** | - | - | - |
|  | Final model: R^2^m=41%, R^2^c=92% | | Final model: R^2^m=38%, R^2^c=95% | | Final model: R^2^m=25%, R^2^c=93% | | - | | Final model: R^2^m=25%, R^2^c=92% | | - | |
| **CGV (mm^3^)** | -4 x 10^-6^ ± 2 x 10^-6^,* | - | 8 x 10^-7^ ± 3 x 10^-7^,* | - | - | - | - | - | 10-4 ± 6 x 10-5,* | - | - | - |
| **Cerebrum (mm^3^)** |  | - | - | - | - | - | - | - | 2 x 10-5 ± 7 x 10-6,*** | - | - | - |
|  | Final model: R^2^m=41%, R^2^c=92% | | Final model: R^2^m=38%, R^2^c=95% | | - | | - | | Final model: R^2^m=25%, R^2^c=92% | | - | |
| **CWV (mm^3^)** | -2 x 10^-5^ ± 6 x 10^-6^,* | - | 3 x 10^-6^ ± 10^-6^,** | - | - | - | - | - | 5 x 10-4, ± 2 x 10-4,** | - | - | - |
| **Cerebrum (mm^3^)** |  | - | - | - | - | - | - | - | 2 x 10-5 ± 7 x 10-6,* | - | - | - |
|  | Final model: R^2^m=41%, R^2^c=92% | | Final model: R^2^m=38%, R^2^c=95% | | - | | - | | Final model: R^2^m=25%, R^2^c=92% | | - | |
| **Ant. lobe (mm^3^)** | -3 x 10^-5^ ± 10^-5^,* | - | 4 x 10^-6^ ± 2 x 10^-6^,* |  | - | - | - | - | - | - | - | - |
| **Cerebrum (mm^3^)** |  | - | - | - | - | - | - | - | - | - | - | - |
|  | Final model: R^2^m=41%, R^2^c=92% | | Final model: R^2^m=38%, R^2^c=95% | | - | | - | | - | | - | |
| **Post. sup. lobe (mm^3^)** | - | - | - | - | - | - | - | - | 2 x 10-4 ± 10-4,* | - | - | - |
| **Cerebrum (mm^3^)** | - | - | - | - | - | - | - | - | 2 x 10-5 ± 7 x 10-6,*** | - | - | - |
|  | - | | - | | - | | - | | Final model: R^2^m=25%, R^2^c=92% | | - | |
| **Post. inf. lobe (mm^3^)** | -7 x 10^-6^ ± 3 x 10^-6^,* | - | 1 x 10-6 ± 5 x 10-7,** | - | - | - | - | - | - | - | - | - |
| **Cerebrum (mm^3^)** |  | - | 7 x 10-8 ± 3 x 10-8,* | - | - | - | - | - | - | - | - | - |
|  | Final model: R^2^m=41%, R^2^c=92% | | Final model: R^2^m=38%, R^2^c=95% | | - | | - | | - | | - | |
| **Lobules IV, VI & VIII (mm^3^)** | n.e. | n.e. | n.e. | n.e. | - | - | - | - | n.e. | n.e. | n.e. | n.e. |
| **Cerebrum (mm^3^)** | n.e. | n.e. | n.e. | n.e. | - | - | - | - | n.e. | n.e. | n.e. | n.e. |
|  | n.e. | | n.e. | | - | | - | | n.e. | | n.e. | |
| **Cerebellar Peduncles (mm^3^)** | n.e. | n.e. | n.e. | n.e. | n.e. | n.e. | n.e. | n.e. | 9 x 10-4 ± 4 x 10-4,** | - | - | - |
| **Cerebrum (mm^3^)** | n.e. | n.e. | n.e. | n.e. | n.e. | n.e. | n.e. | n.e. | 2 x 10-5 ± 2 x 10-6,** | - | - | - |
|  | n.e. | | n.e. | | n.e. | | n.e. | | Final model: R^2^m=26%, R^2^c=92% | | - | |

***Abbreviations:***

*Ant. = anterior, Cerebrum = (supratentorial) cerebral volume, CGV =* *cerebellar grey matter volume, CWV = cerebellar grey matter volume, D9HPT = Dominant-hand 9 hole peg test =, EDSS = Expanded Disability Status Scale, inf. = inferior, ND9HPT = Non-dominanant-hand 9 hole peg test, n.e. = not evaluated, PASAT = Paced Auditory Serial Addition Test, Post. = posterior, R^2^_m_= marginal R-squared; R^2^_c_= conditional R-squared, SDMT = Single Digit Modality Test, T25fwt = Timed 25-foot walk test, TCV = total cerebellar volume.*

*Significance level after correcting using the false discovery rate method: q < 0.05 = *, q < 0.01 = **, q < 0.001 = ****

*Analysis was performed with linear mixed effect models with a random intercept and slope. In all models, independent variables were entered blockwise keeping the following sequence: First demographics (sex, age) and clinical factors (disease duration), then cerebellar volumes and finally supratentorial cerebral volume. Only ipsilateral cerebellar volumes were tested in the analysis of D9HPT and ND9HPT. Each factor was tested both for its contribution to the fit’s intercept as well as to the fit’s slope. All independent variables without statistical significance were excluded from the final model. Boxes display regression coefficients and respective levels of significance.*

**Cerebellar volume changes and prediction of future disability – phase II analyses**

LMER in the whole cohort (Table SM2) showed that all baseline cerebellar volumes but the posterior superior lobe were inversely correlated with the future average EDSS, whereas baseline cerebral volume also contributed in these models. However, no cerebellar metrics were associated with future EDSS changes over time. Average future D9HPT was correlated with the baseline TCV and the annual change rates of the summed volumes of cerebellar lobules IV, VI and VIII. Moreover lower baseline CGV, CWV, posterior superior lobe volume, posterior inferior lobe volume and the summed baseline volumes of cerebellar lobules IV, VI and VIII were by trend associated with future D9HPT worsening over time. The average future ND9HPT was correlated with baseline TCV, posterior superior lobe and by trend with baseline CGV and sum of cerebellar lobules IV, VI and VIII, but no variables were associated with future ND9HPT changes over time. The annual rate of the posterior inferior lobe volume change as well as baseline total cerebellar hemisphere volume, CGV, posterior superior lobe, and the cerebellar peduncles was also correlated to the average future SDMT, but no metrics were associated with future SDMT changes over time. Future T25FWT and PASAT were not correlated with cerebellar volumes.

**Table SM2. Correlations between annual cerebellar volume change rates and future clinical scores in the whole MS cohort.**

| **MRI metrics** | **log(EDSS)** | | **1/T25FWT** | | **log(D9HPT)** | | **log(ND9HPT)** | | **SDMT** | | **PASAT^2^** | |
| --- | --- | --- | --- | --- | --- | --- | --- | --- | --- | --- | --- | --- |
|  | **Average** | **Change over time** | **Average** | **Change over time** | **Average** | **Change over time** | **Average** | **Change over time** | **Average** | **Change over time** | **Average** | **Change over time** |
| **Baseline TCV (mm^3^)** | -2.7x10^-6^±1.7x10^-6^,** | - | - | - | -1.1x10^-5^±4.1x10^-6^,* | - | -8.4x10^-6^±3.3x10^-6^,* | - | 1.3x10^-4^±5.4x10^-5^,* | - | - | - |
| **TCV AVCR (%)** | - | - | - | - | - | - | - | - | - | - | - | - |
| **Baseline Cerebrum (mm^3^)** | -1.1x10^-6^±3.3x10^-7^,** | - | n.e. | n.e. | - | - | - | - | - | - | n.e. | n.e. |
| **Cerebrum AVCR (%)** | - | - | n.e. | n.e. | - | - | - | - | - | - | n.e. | n.e. |
|  | Final model: R^2^m=47%, R^2^c=90% | | - | | Final model: R^2^m=28%, R^2^c=89% | | Final model: R^2^m=33%, R^2^c=90% | | Final model: R^2^m=26%, R^2^c=92% | | - | |
| **Baseline CGV (mm^3^)** | -3.2x10^-6^±2.0x10^-6^,** | - | - | - | - | -1.3x10^-6^±5.0x10^-7^, q=0.057 | -8.9x10^-6^±4.1x10^-6^, q=0.074 | - | 1.6x10^-4^±6.5x10^-5^,* | - | - | - |
| **CGV AVCR (%)** | - | - | - | - | - | - | - | - | - | - | - | - |
| **Baseline Cerebrum (mm^3^)** | -1.1x10^-6^±3.3x10^-7^,** | - | n.e. | n.e. | - | - | - | - | - | - | n.e. | n.e. |
| **Cerebrum AVCR (%)** | - | - | n.e. | n.e. | - | - | - | - | - | - | n.e. | n.e. |
|  | Final model: R^2^m=48%, R^2^c=90% | | - | | Final model: R^2^m=27%, R^2^c=92% | | Final model: R^2^m=26%, R^2^c=94% | | Final model: R^2^m=29%, R^2^c=92% | | - | |
| **Baseline CWV (mm^3^)** | -8.1x10^-6^±7.9x10^-6^,* | - | - | - | - | -4.0x10^-6^±1.9x10^-6^, q=0.064 | - | - | - | - | - | - |
| **CWV AVCR (%)** | - | - | - | - | - | - | - | - | - | - | - | - |
| **Baseline Cerebrum (mm^3^)** | -1.1x10^-6^±3.4x10^-7^,** | - | n.e. | n.e. | - | - | - | - | n.e. | n.e. | n.e. | n.e. |
| **Cerebrum AVCR (%)** | - | - | n.e. | n.e. | - | - | - | - | n.e. | n.e. | n.e. | n.e. |
|  | Final model: R^2^m=47%, R^2^c=90% | | - | | Final model: R^2^m=26%, R^2^c=92% | | - | | - | | - | |
| **Baseline ant. lobe (mm^3^)** | -2.9x10^-5^±1.2x10^-5^,** | - | - | - | - | - | - | - | - | - | - | - |
| **Ant. lobe AVCR (%)** | - | - | - | - | - | - | - | - | - | - | - | - |
| **Baseline Cerebrum (mm^3^)** | -1.0x10^-6^±3.2x10^-7^,** | - | n.e. | n.e. | - | - | - | - | n.e. | n.e. | n.e. | n.e. |
| **Cerebrum AVCR (%)** | - | - | n.e. | n.e. | - | - | - | - | n.e. | n.e. | n.e. | n.e. |
|  | Final model: R^2^m=49%, R^2^c=90% | | - | | - | | - | | - | | - | |
| **Baseline post. sup. lobe (mm^3^)** | - | - | - | - | - | -2.4x10^-6^±1.1x10^-6^, q=0.060 | -2.2x10^-5^±8.2x10^-6^,* | - | 4.2x10^-4^±1.4x10^-4^,* | - | - | - |
| **Post. sup. lobe AVCR (%)** | - | - | - | - | - | - | - | - | - | - | - | - |
| **Baseline Cerebrum (mm^3^)** | n.e. | n.e. | n.e. | n.e. | - | - | - | - | - | - | n.e. | n.e. |
| **Cerebrum AVCR (%)** | n.e. | n.e. | n.e. | n.e. | - | - | - | - | - | - | n.e. | n.e. |
|  | - | | - | | Final model: R^2^m=26%, R^2^c=92% | | Final model: R^2^m=27%, R^2^c=94% | | Final model: R^2^m=30%, R^2^c=92% | | - | |
| **Baseline post. inf. lobe (mm^3^)** | -7.3x10^-6^±3.6x10^-6^,** | - | - | - | - | -2.1x10^-6^±1.0x10^-6^, q=0.064 | - | - | - | - | - | - |
| **Post. inf. lobe AVCR (%)** | - | - | - | - | - | - | - | - | 3.16±1.35,* | - | - | - |
| **Baseline Cerebrum (mm^3^)** | -1.1x10^-6^±3.2x10^-7^,** | - | n.e. | n.e. | - | - | - | - | - | - | n.e. | n.e. |
| **Cerebrum AVCR (%)** | - | - | n.e. | n.e. | - | - | - | - | - | - | n.e. | n.e. |
|  | Final model: R^2^m=48%, R^2^c=90% | | - | | Final model: R^2^m=26%, R^2^c=92% | | - | | Final model: R^2^m=28%, R^2^c=92% | | - | |
| **Baseline Lobules IV, VI & VIII (mm^3^)** | n.e. | n.e. | n.e. | n.e. | - | -2.9x10^-6^±1.3x10^-6^,q=0.057 | -1.7x10^-5^±8.4x10^-6^,q=0.074 | - | n.e. | n.e. | n.e. | n.e. |
| **Lobules IV, VI & VIII AVCR (%)** | n.e. | n.e. | n.e. | n.e. | -0.09±0.03,* |  | - | - | n.e. | n.e. | n.e. | n.e. |
| **Baseline Cerebrum (mm^3^)** | n.e. | n.e. | n.e. | n.e. | - | - | - | - | n.e. | n.e. | n.e. | n.e. |
| **Cerebrum AVCR (%)** | n.e. | n.e. | n.e. | n.e. | - | - | - | - | n.e. | n.e. | n.e. | n.e. |
|  | - | | - | | Final model: R^2^m=29%, R^2^c=90% | | Final model: R^2^m=29%, R^2^c=90% | | - | | - | |
| **Baseline Peduncles (mm^3^)** | n.e. | n.e. | n.e. | n.e. | n.e. | n.e. | n.e. | n.e. | 1.1x10^-3^±5.0x10^-6^,* | - | - | - |
| **Peduncles AVCR (%)** | n.e. | n.e. | n.e. | n.e. | n.e. | n.e. | n.e. | n.e. | - | - | - | - |
| **Baseline Cerebrum (mm^3^)** | n.e. | n.e. | n.e. | n.e. | n.e. | n.e. | n.e. | n.e. | - | - | n.e. | n.e. |
| **Cerebrum AVCR (%)** | n.e. | n.e. | n.e. | n.e. | n.e. | n.e. | n.e. | n.e. | - | - | n.e. | n.e. |
|  | - | | - | | - | | - | | Final model: R^2^m=28%, R^2^c=93% | | - | |

*Abbreviations:*

*AVCR = Annual Volume Change Rate, Ant. = anterior, Cerebrum = (supratentorial) cerebral volume, CGV = cerebellar grey matter volume, CWV = cerebellar grey matter volume, D9HPT = Dominant-hand 9 hole peg test =, EDSS = Expanded Disability Status Scale, inf. = inferior, ND9HPT = Non-dominanant-hand 9 hole peg test, n.e. = not evaluated, PASAT = Paced Auditory Serial Addition Test, Post. = posterior, RRMS = relapsing-remitting MS, R^2^_m_= marginal R-squared; R^2^_c_= conditional R-squared, SDMT = Single Digit Modality Test, sup. = superior T25fwt = Timed 25-foot walk test, TCV = total cerebellar volume.*

*Significance level after correcting using the false discovery rate method: q < 0.05 = *, q < 0.01 = **, q < 0.001 = ****

*Analysis was performed with linear mixed effect models with a random intercept and slope. In all models, independent variables were entered blockwise keeping the following sequence: First demographics (sex, age) and clinical factors (disease duration), then cerebellar volumes and finally supratentorial cerebellar volume. Only ipsilateral cerebellar volumes were tested in the analysis of D9HPT and ND9HPT. Each factor was tested both for its contribution to the fit’s intercept as well as to the fit’s slope. All independent variables without statistical significance were excluded from the final model. Boxes display regression coefficients and respective levels of significance.*
